# Baroreflex Coupling Assessed by Cross-Compression Entropy

**DOI:** 10.3389/fphys.2017.00282

**Published:** 2017-05-10

**Authors:** Andy Schumann, Steffen Schulz, Andreas Voss, Susann Scharbrodt, Mathias Baumert, Karl-Jürgen Bär

**Affiliations:** ^1^Psychiatric Brain and Body Research Group, Department of Psychiatry and Psychotherapy, University Hospital JenaJena, Germany; ^2^Institute of Innovative Health Technologies, Ernst-Abbe-Hochschule Jena, University of Applied Sciences JenaJena, Germany; ^3^School of Electrical and Electronic Engineering, The University of AdelaideAdelaide, SA, Australia

**Keywords:** compression, non-linear dynamics, entropy, symbolization, schizophrenia, handgrip, heart rate, blood pressure

## Abstract

Estimating interactions between physiological systems is an important challenge in modern biomedical research. Here, we explore a new concept for quantifying information common in two time series by cross-compressibility. Cross-compression entropy (CCE) exploits the ZIP data compression algorithm extended to bivariate data analysis. First, time series are transformed into symbol vectors. Symbols of the target time series are coded by the symbols of the source series. Uncoupled and linearly coupled surrogates were derived from cardiovascular recordings of 36 healthy controls obtained during rest to demonstrate suitability of this method for assessing physiological coupling. CCE at rest was compared to that of isometric handgrip exercise. Finally, spontaneous baroreflex interaction assessed by CCE_BRS_ was compared between 21 patients suffering from acute schizophrenia and 21 matched controls. The CCE_BRS_ of original time series was significantly higher than in uncoupled surrogates in 89% of the subjects and higher than in linearly coupled surrogates in 47% of the subjects. Handgrip exercise led to sympathetic activation and vagal inhibition accompanied by reduced baroreflex sensitivity. CCE_BRS_ decreased from 0.553 ± 0.030 at rest to 0.514 ± 0.035 during exercise (*p* < 0.001). In acute schizophrenia, heart rate, and blood pressure were elevated. Heart rate variability indicated a change of sympathovagal balance. The CCE_BRS_ of patients with schizophrenia was reduced compared to healthy controls (0.546 ± 0.042 vs. 0.507 ± 0.046, *p* < 0.01) and revealed a decrease of blood pressure influence on heart rate in patients with schizophrenia. Our results indicate that CCE is suitable for the investigation of linear and non-linear coupling in cardiovascular time series. CCE can quantify causal interactions in short, noisy and non-stationary physiological time series.

## Introduction

One of the most challenging problems in biomedical research is to capture relationships between different physiological subsystems. For example, alterations of the dynamic modulation of heart rate have been found in patients suffering from various diseases (Voss et al., 1995, 1999; Bär et al., 2007b). Revealing possible causes of these pathological changes is of increasing importance for evaluating new therapeutic or diagnostic approaches.

Heart rate and blood pressure are regulated via numerous neural and hormonal feedback mechanisms to respond to changing environments. Various types of pressure- and chemoreceptors collect information from different subsystems of the body. Thus, regulatory interdependencies are rather complex and non-linear (Bär et al., 2007a; Porta et al., 2009). The baroreflex is one of the most powerful mechanisms of short-term heart rate modulation. Pressure receptors detect changes of blood pressure and initiate adaptation of cardiac and vascular function. Immediate influences on heart rate are vagally mediated (Voss et al., 2009). The sympathetic system is also involved in the baroreflex response with an impact on vessel tone, contractility of the myocardium, etc. (Rudas et al., 1999). Thus, the baroreflex can act on various time scales with considerable different delays. Baroreflex sensitivity (BRS) has been used to relate heart rate and blood pressure changes associated with a typical spontaneous baroreflex pattern in a linear fashion.

Cardiac morbidity and mortality is increased in patients with schizophrenia (Ifteni et al., 2014), but underlying causes are not fully understood. In acute schizophrenia, changes in heart rate regulation have been reported in numerous studies (Bär et al., 2005a, 2006, 2007b,c, 2008b; Hingorani et al., 2012). Pathologically altered cardiac modulation most probably contributes to a higher vulnerability to arrhythmias and other severe cardiac events, resulting in an overall elevated cardiac mortality (Bär et al., 2005a, 2007c, 2008b; Ifteni et al., 2014). Besides the vagal influence of breathing on heart rate (respiratory sinus arrhythmia), BRS was demonstrated to be impaired in schizophrenia (Bär et al., 2005a, 2007b, 2008a; Berger et al., 2010; Schulz et al., 2013b, 2014, 2015). Recently, attempts have been made to investigate non-linear properties of cardiovascular function in these patients (Bär et al., 2007b; Schulz et al., 2013b, 2014).

There are various concepts for assessing interaction or coupling in cardiovascular data (Schulz et al., 2013a). According to the definition of causality provided by Wiener, a causal influence on a variable (target) is present, if its forecast improves by knowing another variable (source; Wiener, 1956). Many approaches investigating directional influence rely to some extend on Wiener's principle (Hlavácková-Schindler et al., 2007; Schulz et al., 2013a). First, Granger exploited autoregressive models to evaluate whether predictability of the target's future increases by taking the source signal into account (Granger, 1969; Bressler and Seth, 2011; Faes et al., 2011). Non-linearities and non-stationarities complicate the investigation of real physiological data and can lead to an inadequate estimation of Granger causality (Granger and Newbold, 1974; Bressler and Seth, 2011). Concepts borrowed from information theory have gained importance for describing the variability of physiological time series and to estimate their interrelation (Voss et al., 1995, 2009; Schreiber, 2000; Gourévitch et al., 2006; Hlavácková-Schindler et al., 2007; Hlavácková-Schindler, 2011; Javorka et al., 2011).

Here, we introduce cross-compression entropy (CCE), that incorporates the principle idea of causality by adaption of a data compression technique based on symbol transformation. CCE exploits a popular string compression algorithm implemented in common tools, like *zip*-archiving (Ziv and Lempel, 1977; Baumert et al., 2004). Its application to biomedical data was first proposed by Nagarajan (2002). The compressibility of heart rate as a measure of short-term modulation allows forecasting of arrhythmias and risk stratification (Baumert et al., 2004; Truebner et al., 2006; Voss et al., 2008), and has also been investigated in healthy subjects and non-cardiac diseases (Bär et al., 2007b, 2009, 2012; Javorka et al., 2008; Schulz et al., 2010). Compression entropy estimates to which extent a time series can be reproduced by its own past by comparing symbols in a lookahead buffer with recently encoded symbols. We modified this approach to analyze common symbol sequences in bivariate data sets. If a symbol pattern in the target's lookahead buffer occurs also in the memory of the source series but not in the target's own memory, the target can be represented by fewer symbols knowing the source series. This compression improvement can be interpreted as a reduction of uncertainty about the target's future.

The main objective of this study was to investigate whether CCE is capable of quantifying baroreflex sensitivity. Therefore, we synthesized surrogate data from cardiovascular data recorded during rest. Additionally, we analyzed the effect of isometric handgrip exercise on CCE in healthy subjects and compared patients with acute schizophrenia to healthy subjects at rest.

## Materials and methods

### Data recording and preprocessing

#### Study 1: healthy controls

We conducted cardiovascular recordings in 38 right-handed healthy volunteers at rest and during isometric exercise in supine position. First, the maximum voluntary contraction (MVC) was estimated. Subjects were asked to press a hand clench manometer (handgrip, TSD121B, BIOPAC Systems Inc., Goleta, CA, USA) with the maximum affordable power for 30 s. The highest force achieved in three attempts was stored as individual MVC (Iellamo et al., 1999). After 15 min baseline recording at rest, subjects performed an isometric handgrip maneuver maintaining 25% of MVC over 5 min. Target and actual power were displayed via a monitor fixed over the couch. Electrocardiogram (ECG) and non-invasive blood pressure were recorded simultaneously by the MP150 system (BIOPAC Systems Inc., Goleta, CA, USA). Both signals were band pass filtered from 0.05 to 35 Hz and digitized at 1,000 Hz. This hardware filter setting is recommended by Biopac Systems (e.g., in application notes 109 and 233, available at www.biopac.com) in order to reduce high frequency distortions (e.g., power line frequency of 50 Hz) and baseline drifts. Heart beat intervals (BBI series) were extracted from the ECG, checked by manual inspection and preprocessed by adaptive filtering (Wessel et al., 2000). Bivariate cardiovascular data sets were built from BBI and systolic blood pressure values (SBP) embedded in each heart cycle. The data of two participants was excluded from the analysis due to movement artifacts in the ECG. The final group comprised 36 subjects (21 females, 15 males, age: 25.7 ± 4.7 years, BMI: 22.8 ± 3.1). To allow participants to adjust to the environment, we excluded the first 5 min of the recording from the analysis. The last 4 min of the handgrip exercise were extracted and compared to rest. We wanted to avoid any potential influence of different data sizes in both conditions on CCE estimation. Thus, the same number of heart cycles was extracted from the end of the resting state recording as analyzed during exercise (average length of time series was *N*_0_ = 292 ± 43 samples).

#### Study 2: acute schizophrenia

ECG and non-invasive blood pressure were recorded in 21 patients with acute schizophrenia (9 females, 12 males, age: 31.5 ± 2.3 years, BMI: 24.5 ± 1.0) and 21 matched healthy controls (9 females, 12 males, age: 32.1 ± 2.6 years, BMI: 23.8 ± 0.9). Examinations were conducted at rest for 30 min in supine position in a quiet room. The Task Force Monitor system digitized ECG at 1,000 Hz and automatically extracted heart beats and blood pressure values from the raw signals. Resulting time series were preprocessed by adaptive filtering (Wessel et al., 2000). We refer to Bär et al. for details on data acquisition and patients' characteristics (Bär et al., 2007a).

Both studies were approved by the Ethics Committee of the Medical Faculty of the Friedrich-Schiller-University Jena (Ethik-Kommission). All participants gave their informed written consent in accordance with the Declaration of Helsinki. Neither patients nor controls suffered from any medical or additional psychiatric disease, and none of them was receiving any interfering medication that might affect cardiac autonomic function. Diagnosis of paranoid schizophrenia was established by a staff psychiatrist when symptoms of patients who were admitted to our inpatient wards fulfilled DSM-IV criteria (Diagnostic and Statistical Manual of Mental Disorders, 4th edition, published by the American Psychiatric Association). Healthy control subjects were checked for neurological, psychiatric or other clinically significant disorders.

### Cross-compression entropy (CCE)

**Figure 1:**
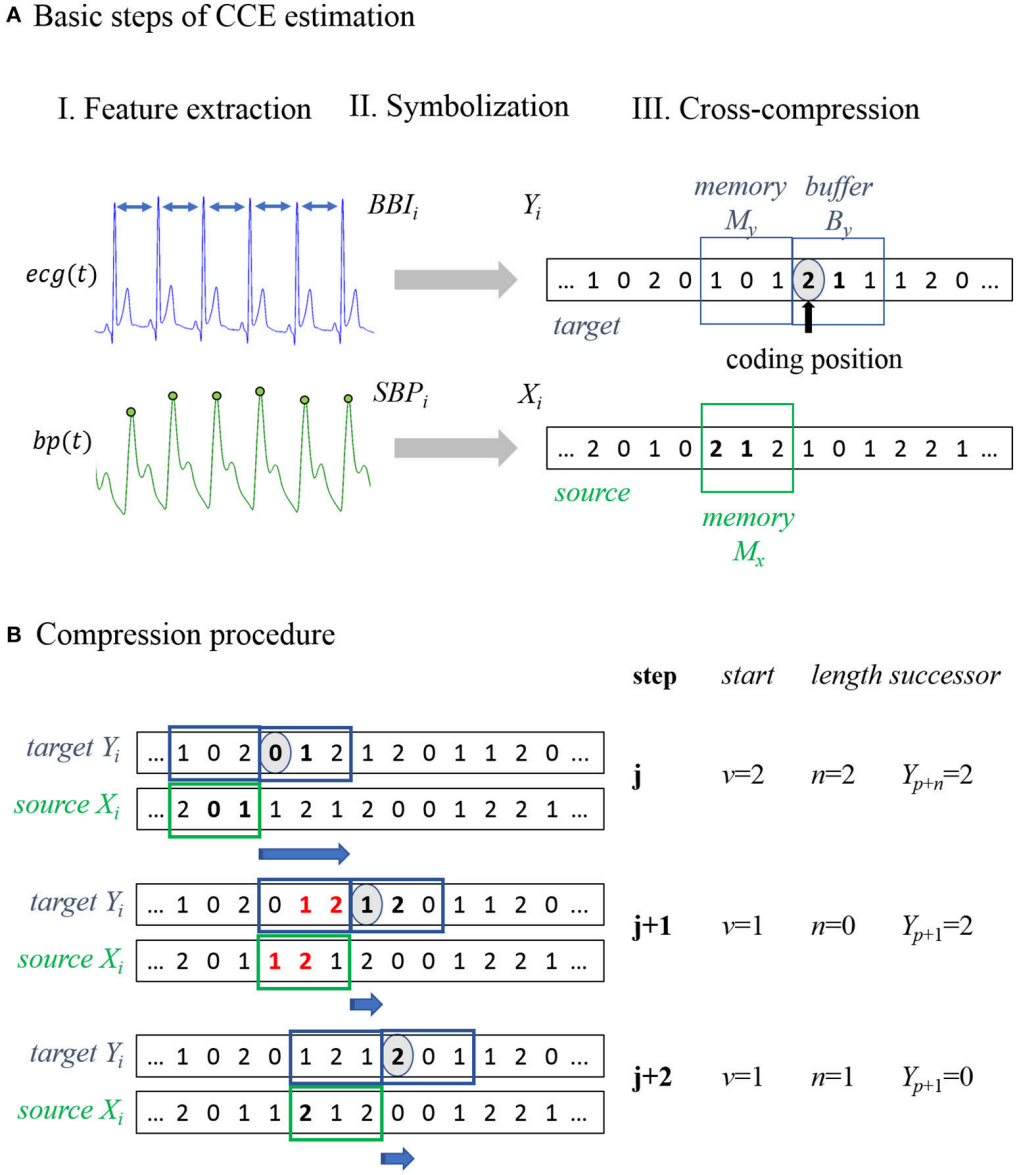
**Schematic illustration of cross-compression entropy (CCE) estimation. (A)** Overview of the three basic steps to calculate CCE. I. Feature extraction: Beat-to-beat intervals BBI and systolic blood pressure SBP were extracted from the recorded signals (ecg, electrocardiogram; bp, blood pressure). II. Symbolization: Time series were transformed to symbol series *X*_*i*_ and *Y*_*i*_. III. Cross-compression: Symbols of *Y*_*i*_ in the target window are encoded using the symbols of *X*_*i*_ in the source window. **(B)** Compression procedure of example symbol series: in step *j*, a substring [0;1] in the target buffer *Y*_*B*_ = [0;1;2] occurs in the source memory *X*_*M*_ = [2;0;1] beginning at second place (*v* = 1) with a length of two symbols (*n* = 2). Redundant symbols (written in bold) can be skipped, storing start *v*, length *n*, and the next symbol *Y*(*n*+*p*). In step j+1, a target substring *Y*_*B*_ = [1;2;0] of two symbols [1;2] occurs in the source memory *X*_*M*_ = [1;2;1]. Because a string of the same length that matches buffer symbols, is included in the target memory, compression is not enhanced by considering the source. Therefore, the redundant strings are ignored (bold and red symbols) and the next symbol stored is *Y*(*p*+1). In the last step (j+2), no string with *n* > 1 was found and all windows were shifted forth in time by one sample *Y*(*p*+1).

The basic steps to estimate CCE are illustrated in Figure 1A. In the preprocessing procedure, characteristic values were extracted from the recorded biosignals (Figure 1A, I Feature extraction). The input time series were transformed into sequences of symbols (e.g., *x*_*i*_ to *X*_*i*_). Recently, Schulz et al. proposed an appropriate symbolization of cardiovascular data applying the thresholds known from the dual sequence method, *T*_*BBI*_ = 5 ms and *T*_*SBP*_ = 1 mmHg (Malberg et al., 1999; Voss et al., 1999; Schulz et al., 2013b). We adopted these thresholds *T*_*x*_ transforming the input into three symbols in order to differentiate distinct tachy- and brady-cardiac influences form minor heart rate fluctuations.

$$\begin{array}{l} X_{i}=\{\begin{array}{ll} 2; & x_{i}-x_{i-1}>T_{x}\\ 1; & |x_{i}-x_{i-1}|\leq T_{x}\\ 0; & x_{i}-x_{i-1}<-T_{x} \end{array} \end{array}$$

The original compression procedure by Ziv and Lempel is extensively described elsewhere (Ziv and Lempel, 1977; Baumert et al., 2004). In the following, we briefly outline the idea modified to analyze physiological signals (Nagarajan, 2002; Baumert et al., 2004, 2005b). The encoding process is conducted in two adjacent time windows shifted along the input. One filled with already encoded symbols (memory) and one covering the current data point and subsequent symbols (buffer). If the current substring to be encoded (stored in the buffer) appears in the memory, it can be skipped storing just the start and length of the redundant memory string. Thus, the number of iterations needed to encode the input can be reduced. Applying this procedure, self-similar input strings can by compressed without losing information.

In the bivariate approach, the target series *Y*_*i*_ is compressed regarding the symbols of the source series *X(i)* (see Figure 1A, III Cross-compression). In addition to the target buffer and memory, a source memory window is defined. In Figure 1, all windows have a length of three samples. Target symbols in the buffer that also occur in the source memory can be skipped to reduce the length of the compressed target series. If there is a redundant substring, of the same length or longer, included in the target memory, the matching source string is ignored because compressibility is not improved by taking the source signal into account. In this way, cross-compression is conditioned on self-compressibility. The application of this procedure to an example symbol series is illustrated and described in Figure 1B. In the following, subsequences (e.g., of the input series *X*_*i*_) from element *k* to *l* are denoted as $X_{k}^{l}=\left[X_{k},X_{k+1},\ldots ,X_{l}\right]$.

The target buffer window covers *B*_*y*_ symbols $Y_{p}^{p+B_{y}}$, starting at the current data point *Y*_*p*_ (coding position). These target symbols are encoded using the symbols of the source memory window $X_{p-M_{x}}^{p-1}$ with length *M*_*x*_. The longest subseries $X_{p-M_{x}+v-1}^{p-M_{x}+v+n-2}$, lasting *n* source symbols starting at element *v*, that matches the target sequence $Y_{p}^{p+n-1}$ is extracted. Instead of encoding the whole target string, the starting point *v* and the length *n* of its equivalent in the source memory and successor *X*_*p*+*n*_ is stored. Hence, *n* target symbols can be passed and the new coding position is *X*_*p*+*n*+1_.

If there is a substring $Y_{p-M_{y}+k-1}^{p-M_{y}+k+l-2}$ with a length of *l* ≥ *n* in the target memory that matches $Y_{p}^{p+l-1}$, the target symbols were compressed equally or even more efficiently by the target's own memory (self-compression). In this situation, the matching source symbols were ignored (*n* = 0) and the next symbol to be encoded was *X*_*p*+1_. The compression procedure can be described by the following stepwise instructions:

*Step 1*: Define analysis windows initially.
*Step 2*: Find longest substring (of length *n*) in the source memory that matches the target buffer string.
*If n* ≤ 1: Store *v* (*v* = 0), *n* and *Y*(*p*+1); shift all windows by 1 sample and go to *step2*.
*If n* > 1: Proceed with *step 3*.
*Step 3*: Find longest substring (of length *l*) in the target memory that matches the target buffer string.
*If l* ≥ *n*: Store *v* (*v* = 0), *n* (*n* = 0) and *Y*(*p*+1), shift all windows by 1 sample and go to *step 2*.
*If l* < *n*: Store *v, n* and *Y*(*p*+*n*), shift all windows by *n*+1 samples and go to *step 2*.

We defined *CCE*_*X*→*Y*_ as the proportion of iterations that can be saved compressing *Y*_*i*_ by *X*_*i*_ with respect to the original length of *Y*_*i*_. Thus, CCE rises with increasing amount of information about the target series recurring in the source series. Whenever a redundant substring, of length *n* > 1 and *n* > *l*, is found in the source memory and target buffer, the number of steps needed to encode the target (*N*_*com*_) is reduced by *n*. Assuming the input *Y*_*i*_ of length *N*_0_ is compressed by *X*_*i*_ in *N*_*com*_ iterations, CCE is calculated by the equation below:

$$\begin{array}{l} CCE_{X\to Y}=\frac{N_{0}-N_{com}}{N_{0}} \end{array}$$

CCE is dependent on the length of the target memory window (*M*_*y*_), the target buffer window (*B*_*y*_), and the source memory window (*M*_*x*_). We defined both memory windows to have the same length (*M*_*y*_ = *M*_*x*_) and reported only *M*_*x*_ in the following analyses. Additionally, we introduced a shift of the source window forth in time, overlap τ, to allow temporally overlapping sequences in both windows. Possible starting points *v* of matching sequences in the source window were restricted to *v* < *M*_*x*_−τ+1. Thus, these sequences found in the target window cannot temporal precede the respective pattern in the source window. CCE assesses redundant information with several time lags. If overlap τ > 0, all delays *d* with 0 ≤ *d* ≤ *M*_*x*_−τ contribute to CCE estimation (1 ≤ *d* ≤ *M*_*x*_ for τ = 0).

To estimate baroreflex modulation of heart rate induced by changes of blood pressure, we defined BBI as the target signal *Y*_*i*_ and SBP as the source signal *X*_*i*_. Considering the established methods, e.g., joint symbolic dynamics JSD and the dual sequence method (see below), baroreflex activity is likely expressed by patterns of at least three consecutive, symmetric changes of BBI and SBP. Delays from zero to three heart cycles were found to be physiologically meaningful (Voss et al., 1999). We derived one preset from this prior knowledge and calculated CCE_BRS_ using *M*_*x*_ = 4, *B*_*y*_ = 4, and τ = 3. CCE_BRS_ captures redundant symbol strings of lengths up to four symbols and delays from zero and one sample. In study 1, we additionally analyzed the influence of estimation settings on CCE results for *M*_*x*_ = {3,4,5,7,10}, *B*_*y*_ = {3,4,5,7,10}, and τ = {0,1,3}.

### Baroreflex sensitivity (BRS)

Sensitivity of the baroreflex was quantified using the dual sequence method (DSM; Malberg et al., 1999). The pattern of spontaneous bradycardiac baroreflex regulation was defined as three consecutive BBI and SBP synchronously increasing by at least 5 ms and 1 mmHg, respectively. Linear regression was performed on the three SBP-BBI-pairs of each sequence. The mean slope was used as index of baroreflex sensitivity (BRS_b_) given in ms/mmHg. Besides this classical bradycardiac influence, a tachycardiac effect can be evaluated analyzing patterns of decreasing SBP and BBI values (BRS_t_; Voss et al., 1999; Bär et al., 2007a).

### Joint symbolic dynamics (JSD)

The methodology of joint symbolic dynamics for analysis of cardiovascular coupling was introduced by Baumert et al. (2002). The time series of heart beat intervals and systolic blood pressure values were transformed into symbol sequences differentiating positive and non-positive changes. Subsequences of three symbols were extracted (words). Relative frequencies of joint occurrences of these words were represented by an 8 × 8 word distribution matrix. The probabilities of all symmetric and diametric word types were extracted from the matrix by summing up all diagonal (JSD_sym_) and counter diagonal elements (JSD_diam_), respectively (Baumert et al., 2005a). JSD_sym_ can be interpreted as proportion of baroreflex-related patterns on the whole recording. The algorithm was applied to cardiovascular data in various studies (Baumert et al., 2005b, 2013a; Wessel et al., 2009; Suhrbier et al., 2010).

### Standard indices of heart rate variability and blood pressure

Mean heart rate (HR), systolic and diastolic blood pressure (SBP, DBP) were estimated by averaging the respective time series. Standard deviation of heart beat intervals (sdNN), root mean square of successive interval differences (RMSSD), and ratio of low to high frequency power (LF/HF) were calculated to evaluate global and short-term HR variability, and cardiac sympathovagal balance (Malik et al., 1996). Univariate compression entropy (Hc_BBI_) was estimated following the original algorithm described by Baumert with recommended window lengths *w* = 7, *b* = 3 (Baumert et al., 2004; Truebner et al., 2006). Additionally, we calculated compression entropy of BBI transformed by Equation (1) into three symbols (Hc_Y_) according to CCE preprocessing with *w* = 7, *b* = 3.

### Surrogate data

The surrogate data approach (Theiler et al., 1992; Schreiber and Schmitz, 2000) was applied to test the significance and the non-linear nature of CCE of BBI by SBP (Schulz et al., 2013b).

- Uncoupled isospectral isodistribution pairs (sI) from the original BBI and SBP time series were created to preserve linear properties to test for coupling. Surrogate data sI have the same frequency distribution and power spectra as the original pairs of signals, but phases were taken randomly from uniform distribution (0–2π). Inverse amplitude-adjusted Fourier transform (IAAFT) was used to preserve distribution of the original series and approximate the original power spectrum.
- Isospectral isodistribution pairs (sII) from the original BBI and SBP time series were created preserving cross-correlation to test for coupling-non-linearity. These surrogate data preserved the individual BBI and SBP spectra (IAAFT) as well as the magnitude of their cross-spectrum obtained by adding the same random number to the Fourier phases of the two series. Thus, the linear coupling was maintained, whereas non-linear interactions were destroyed (Nollo et al., 2002).

### Data analysis

Synthesized data was used to evaluate if CCE captures the physiological cardiovascular coupling (sI) and to which extent this coupling is non-linear (sII). Twenty independent surrogate data sets of type sI and sII were derived from each of the resting state time series in study 1 (*n* = 36).

Assuming the physiological coupling of original cardiovascular time series to be destroyed in surrogates sI, we expect smaller CCE results in the surrogate data. In accordance to the probability of error *p* = 5%, we considered CCE of one subject “significant,” i.e., confirming CCE to reflect true interaction, if at most one of the surrogates revealed a higher CCE than the original data. In the same way, we tested in how many subjects CCE revealed significant non-linear coupling using surrogates sII.

Wilcoxon signed rank test for paired samples was conducted for comparison of parameters estimated during rest and exercise. Statistical significance was assumed at commonly used *p*-value levels: ^*^p < 0.05; ^**^p < 0.01; ^***^p < 0.001.

In study 2, independent samples of healthy controls and patients suffering from acute schizophrenia were compared by non-parametric rank sum test.

All analyses were performed in MATLAB (version 2012a, The Mathworks Inc., Natick, MA, USA) and SPSS (version 23, IBM, Chicago, IL, USA). Descriptive statistics are reported in mean values ± standard deviation. Linear dependencies were analyzed by spearman correlation coefficient *r* and are reported together with asterisks representing the estimated *p*-values (^*^p < 0.05; ^**^p < 0.01).

## Results

### Study 1: healthy controls

**Figure 2:**
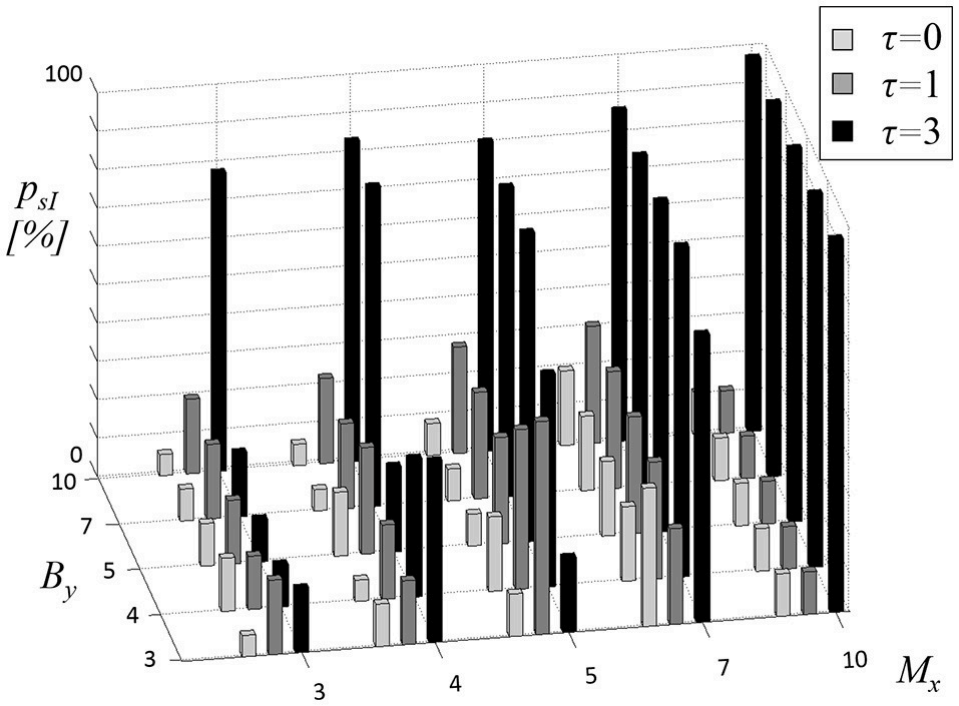
**Proportion ***p***_***sI***_ of subjects with significant cross-compression entropy (CCE) results compared to uncoupled surrogates (sI)**. The length of source memory window *M*_*x*_ (equal to target memory window length *M*_*y*_), the length of target window *B*_*y*_ and the overlap τ (gray shades of bars) were varied.

First, CCE was tested to reveal physiological coupling by comparing resting state cardiovascular recordings to uncoupled surrogate data sets. In Figure 2, the proportion *p*_*sI*_ of subjects with significant CCE is presented. The results were explicitly dependent on the parameters *M*_*x*_, *B*_*y*_, and τ. The overlap seems to influence the results systematically by limiting the maximum amount of significant CCE. For τ = 0 (light gray bars in Figure 2) the highest proportion *p*_*sI*_ = 36% was calculated using *M*_*x*_ = 3 and *B*_*y*_ = 4. There was no obvious linear dependency of *p*_*sI*_ on the two window lengths *M*_*x*_, *B*_*y*_. With an overlap of one sample (τ = 1, dark gray bars in Figure 2), the highest amount of significant results *p*_*sI*_ = 56% was estimated applying *M*_*x*_ = 3 together with *B*_*y*_ = 5. The percentage seemed not to be proportional to either of the window sizes *M*_*x*_, *B*_*y*_. Using τ = 3, *p*_*sI*_ was maximized to 97% in combination with *M*_*x*_ = 3, irrespective of the choice of *B*_*y*_ (black bars in Figure 2). With an overlap τ = 3, high *p*_*sI*_-values (*p*_*sI*_ ≥ 81%) were found when the window sizes were equal (*M*_*x*_ = *B*_*y*_). Enlarging the target window to *B*_*y*_ > *M*_*x*_ did not affect *p*_*sI*_ because the buffer string was cut to the size of the memory window (*n*_*max*_ = *M*_*x*_). Thus, in the following analyses, the length of the target window was limited to *B*_*y*_ ≤ *M*_*x*_.

The parameter most influential on *p*_*sI*_ appeared to be overlap τ. It was adjusted to three samples (τ = 3) to maximize the number of significant CCEs compared to uncoupled surrogates. Numerical values of the results depicted in Figure 2 (black bars) are reported in Table 1 (left column). In the right column of Table 1 the proportion *p*_*sII*_ of significant CCE results compared to linearly coupled surrogates sII are listed (τ = 3).

**Table 1:** **Proportion of subjects with significant cross-compression entropy (CCE) results compared to uncoupled sI and linearly coupled surrogates sII**.

**Uncoupled surrogate data (sI)**						**Linearly coupled surrogate data (sII)**					
***B_y_\M_x_***	**10**	**7**	**5**	**4**	**3**	***B_y_\M_x_***	**10**	**7**	**5**	**4**	**3**
3	19%	50%	22%	78%	97%	3	14%	22%	3%	33%	64%
4	14%	39%	58%	**89%**		4	6%	17%	22%	**47%**	
5	14%	25%	83%			5	14%	8%	42%		
7	19%	86%				7	6%	44%			
10	81%					10	53%				

*Lengths of the source memory window (with M_y_ = M_x_) and the target window B_y_ varied from 3 to 10 samples. The overlap was adjusted to τ = 3 samples. For B_y_ > M_x_ CCE results were not affected by increase of B_y_. Bold values correspond to the a priori defined parameter set (CCE_BRS_)*.

Compared to uncoupled surrogates sI, the highest amount of significant CCE results of *p*_*sI*_ = 97% was achieved using window lengths of *M*_*x*_ = *B*_*y*_ = 3 and an overlap of τ = 3. This is in accordance to JSD analyses of synchronous patterns consisting of three symbols. Using these settings, original CCE exceeded linearly coupled surrogates in *p*_*sII*_ = 64% of the subjects, which was the maximum in sII-analysis. The a priori determined preset (*M*_*x*_ = 4, *B*_*y*_ = 4, and τ = 3) revealed the second highest proportion for each of the two types of surrogate data (*p*_*sI*_ = 89%, *p*_*sII*_ = 47%, bold elements in Table 1).

During exercise condition, mean heart rate and both blood pressure indices increased significantly (see Table 2). Parameters of heart rate variability were reduced with RMSSD and Hc_BBI_ at a high significance level (*p* < 0.001). Baroreflex sensitivity with bradycardiac and tachycardiac influence decreased during handgrip maneuver (BRS_b_: *p* < 0.05, BRS_t_: *p* < 0.001). JSD_sym_ and JSD_diam_ were not changed significantly by exercise intervention. CCE_BRS_ fell from 0.553 at rest to 0.514 (*p* < 0.001). Compression entropy of symbolized heart beat intervals Hc_Y_ was higher during handgrip (*p* < 0.001).

**Table 2:** **Results of signed rank test of autonomic indices during rest vs. exercise condition**.

**Parameter**	**Resting state**	**Exercise**	**Significance**
**CARDIOVASCULAR INDICES**			
HR (min^−1^)	64.54 ± 9.59	73.73 ± 10.88	^***^
LF/HF	1.58 ± 1.73	2.13 ± 2.22	n.s.
sdNN (ms)	65.87 ± 23.24	60.61 ± 28.04	n.s.
RMSSD (ms)	58.85 ± 27.12	41.40 ± 24.41	^***^
Hc_BBI_	0.903 ± 0.054	0.875 ± 0.036	^*^
Hc_Y_	0.258 ± 0.001	0.269 ± 0.003	^***^
SBP (mmHg)	112.31 ± 12.09	120.56 ± 15.03	^***^
DBP (mmHg)	69.80 ± 9.52	80.23 ± 10.97	^***^
**BAROREFLEX INDICES**			
BRS_b_ (ms/mmHg)	19.39 ± 10.72	14.93 ± 10.32	^*^
BRS_t_ (ms/mmHg)	19.67 ± 10.10	13.56 ± 8.43	^***^
JSD_sym_	0.241 ± 0.082	0.216 ± 0.088	n.s.
JSD_diam_	0.056 ± 0.039	0.061 ± 0.031	n.s.
CCE_BRS_	0.553 ± 0.030	0.514 ± 0.035	^***^

*Results given in mean ± standard deviation. HR, heart rate; sdNN, standard deviation of normal heart beat intervals; RMSSD, root mean square of successive heart beat intervals; SBP, systolic blood pressure; DBP, diastolic blood pressure; BRS_b_, bradycardiac baroreflex sensitivity; BRS_t_, tachycardiac baroreflex sensitivity; JSD_sym_, joint symbolic dynamics symmetric patterns; JSD_diam_, joint symbolic dynamics diametric patterns; CCE_BRS_, cross-compression entropy (M_x_ = M_y_ = 4, B_y_ = 4 and τ = 3); Hc_BBI_, compression entropy (w = 3, t = 7, no overlap); Hc_Y_, compression entropy of symbolized heart rate (w = 3, t = 7, no overlap)*.

* p < 0.05;

*** *p < 0.001*.

At rest, CCE_BRS_ was exclusively correlated to JSD_sym_ (*r* = 0.43^**^). During handgrip, CCE_BRS_ was correlated positively to RMSSD (0.54^**^), BRS_b_ (0.44^**^), and BRS_*t*_ (0.36^*^) and negatively to HR (*r* = −0.63^*^) and LF/HF (*r* = −0.60^**^). The relation of CCE_BRS_ to JSDsym remained during exercise (JSD_sym_: *r* = 0.43^**^).

In Table 3, differences of CCE results at rest vs. exercise are depicted. All parameter settings revealed significant decreases of CCE during exercise. Using the CCE_BRS_-presets *M*_*x*_ = *B*_*y*_ = 4 (and τ = 3) the mean decrease was second largest and highly significant (−0.038 ± 0.042, *p* < 0.001).

**Table 3:** **CCE differences at rest vs. exercise**.

***B_y_$\tilde{M}$_x_***	**10**	**7**	**5**	**4**	**3**
3	−0.014 ± 0.027^**^	−0.012 ± 0.029^*^	−0.012 ± 0.026^*^	−0.017 ± 0.028^**^	−0.038 ± 0.041^***^
4	−0.015 ± 0.026^**^	−0.017 ± 0.029^**^	−0.015 ± 0.03^*^	−**0.038** ± 0.042^***^	
5	−0.009 ± 0.022^*^	−0.027 ± 0.035^***^	−0.039 ± 0.041^***^		
7	−0.029 ± 0.031^***^	−0.037 ± 0.04^***^			
10	−0.037 ± 0.045^***^				

Results are given in mean values ± standard deviation. Statistical significance was computed by signed rank test. Asterisks were attached to significant differences (

* p < 0.05;

** *p < 0.01*,

*** *p < 0.001)*.

*The target memory window has the same size as the source memory window (M_y_ = M_x_). Previously defined CCE_BRS_ with M_x_ = M_y_ = 4, B_y_ = 4 and τ = 3 was written in bold letters*.

### Study 2: acute schizophrenia

As depicted in Table 4, patients with acute schizophrenia had an elevated mean heart rate (*p* < 0.001) and blood pressure (*p* < 0.01). Heart rate variability was reduced in patients (sdNN and Hc_BBI_, *p* < 0.01). Increased LF/HF ratio (*p* < 0.05) and diminished RMSSD (*p* < 0.001) indicated a sympathetic predominance of heart rate modulation. In contrast, compression entropy of symbolized heart beat intervals (Hc_Y_) was almost identical in both groups (0.252 vs. 0.253).

**Table 4:** **Results of rank sum test of autonomic indices in healthy subjects vs. acute schizophrenia**.

**Parameter**	**Healthy controls**	**Schizophrenia**	**Significance**
**CARDIOVASCULAR INDICES**			
HR (min^−1^)	65.17 ± 5.29	81.18 ± 11.89	^***^
LF/HF	1.29 ± 1.23	2.31 ± 2.20	^*^
sdNN (ms)	85.52 ± 31.59	56.76 ± 25.77	^**^
RMSSD (ms)	58.97 ± 25.77	31.1 ± 18.18	^***^
Hc_BBI_	0.798 ± 0.060	0.728 ± 0.102	^**^
Hc_Y_	0.253 ± 0.001	0.252 ± 0.001	n.s.
SBP (mmHg)	120.48 ± 10.34	134.66 ± 14.09	^**^
DBP (mmHg)	73.64 ± 8.28	84.08 ± 11.67	^**^
**BAROREFLEX INDICES**			
BRS_b_ (ms/mmHg)	22.47 ± 12.02	11.15 ± 9.30	^***^
BRS_t_ (ms/mmHg)	18.41 ± 9.45	13.84 ± 7.26	^***^
JSD_sym_	0.437 ± 0.071	0.262 ± 0.106	^***^
JSD_diam_	0.016 ± 0.012	0.056 ± 0.038	^***^
CCE_BRS_	0.546 ± 0.042	0.507 ± 0.046	^**^

*Results given in mean ± standard deviation. HR, heart rate; sdNN, standard deviation of normal heart beat intervals; RMSSD, root mean square of successive heart beat intervals; SBP, systolic blood pressure; DBP, diastolic blood pressure; BRS_b_, bradycardiac baroreflex sensitivity; BRS_t_, tachycardiac baroreflex sensitivity; JSD_sym_, joint symbolic dynamics symmetric patterns; JSD_diam_, joint symbolic dynamics diametric patterns; CCE_BRS_, cross-compression entropy (M_x_ = M_y_ = 4, B_y_ = 4 and τ = 3); Hc_BBI_, compression entropy (w = 3, t = 7, no overlap), Hc_Y_, compression entropy of symbolized heart rate (w = 3, t = 7, no overlap)*.

* p < 0.05;

** *p < 0.01*,

*** *p < 0.001*.

All baroreflex indices revealed impaired cardiovascular regulation in patients with schizophrenia. Bradycardiac baroreflex sensitivity in controls was twice as high when compared to controls (*p* < 0.001). Indices of joint symbolic dynamics were shifted from symmetric to diametric patterns in schizophrenia, demonstrating a decreased occurrence of baroreflex patterns (*p* < 0.001). CCE_BRS_ indicated a loss of compressibility of heart rate by blood pressure in patients (*p* < 0.01).

**Figure 3:**
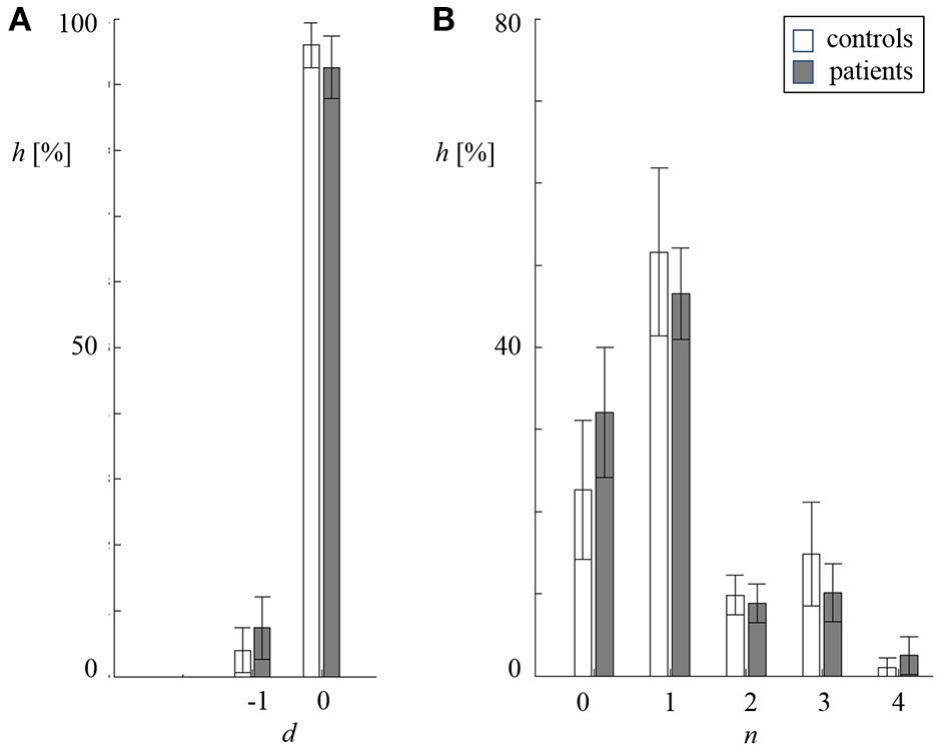
**Distribution of delay ***d*** (A) and length *n***(B)** of redundant symbol patterns during cross-compression** (standard deviation indicated by error bars, white: controls, gray: patients). **(A)** Percentage frequency *h* of delays *d* being zero and minus one sample. **(B)** Percentage frequency *h* of lengths *n* being 0–4 samples.

In Figure 3, the distribution of the delay *d* and length *n* of redundant symbol strings during cross-compression are depicted. Starting position *v* was converted into the respective delay *d* = τ−*M*_*x*_+*v*. In both groups, primarily synchronous patterns (*d* = 0) contributed to CCE. The length of symbol patterns *n* varied from 0–4 samples, where *n* = 0 means that either no symbols occurred in both the source memory and target buffer, or a redundant string of at least the same length was found in the target memory (see Figure 1B). Single recurring symbols (*n* = 1) were found most frequently, but only substrings with *n* ≥ 2 improve cross-compression effectively. The average probability to find redundant strings of length *n* = 0 and *n* = 4 was higher in patients with schizophrenia.

In the group of healthy controls, CCE_BRS_ was correlated to both JSD parameters (JSDsym *r* = 0.66^**^, JSD_diam_ *r* = −0.47^*^). In patients with acute schizophrenia, cardiac indices HR (*r* = −0.50^**^) as well as symbolic metrics JSD_sym_ (*r* = 0.53^**^) and JSD_diam_ (*r* = −0.61^**^) correlated to CCE_BRS_.

## Discussion

The main objective of this work was to introduce CCE and to evaluate whether it can quantify the strength of baroreflex influence on heart rate. We defined a preset for CCE_BRS_ estimation based on previous assumptions, and investigated CCE's dependence on various parameter settings. Surrogate analysis revealed that CCE_BRS_ captures physiologically meaningful cardiovascular coupling that is both linear and non-linear. CCE_BRS_ was significantly reduced in controls during exercise and in patients with acute schizophrenia at rest when compared to healthy subjects at rest.

In study 1, we investigated the influence of parameter settings on CCE estimation. *A priori*, one preset was determined for the assessment of baroreflex interaction. CCE_BRS_ focuses on a rapid coupling mechanism, as we use short window lengths and a large overlap. Interaction delays defined by that preset were zero and one heart cycle. Thus, it can be assumed that only parasympathetic influence contributes to CCE_BRS_. Surrogate analysis sI with varying window sizes *M*_*x*_ and *B*_*y*_ confirmed that cardiovascular coupling occurs on different time scales. Interaction delays covered by CCE analysis, ranged from zero to seven samples and length of redundant information ranged from 2 to 10 samples.

The comparison to linearly coupled surrogates sII indicated that especially instantaneous interaction, i.e., when the source window was as small as the overlap (*M*_*x*_ = τ), is of non-linear nature. Regulatory mechanisms of this temporal dynamic react promptly to sudden environmental changes. Heart rate (HR) itself has an immediate influence on blood pressure. Several feedback loops are involved in HR modulation. For example, respiratory sinus arrhythmia acts on HR due to changes of breathing. This, in turn, affects blood pressure. Thus, respiratory rhythms can be found in the cardiovascular system as well. The variety of interacting physiological mechanisms and the need for immediate responses may explain the non-linear nature of short-term coupling.

During exercise, the metaboreflex was initiated by metabolites accumulated in the contracting muscles (Goodwin et al., 1972). The elevation of efferent sympathetic nerve activity leads to an increase of systemic vessel tone and has inotropic and chronotropic impacts on the heart (Boushel, 2010). The *central command* is supposed to facilitate the cardiac sympathetic activation by parasympathetic withdrawal (Boushel, 2010; Fisher et al., 2010). CCE decrease using all calculation settings corroborates changes of cardiovascular coupling across all resulting time scales (Iellamo et al., 1999; Fisher et al., 2010). We found an increase of mean HR, systolic and diastolic blood pressure and diminished short-term HR fluctuations (RMSSD and Hc_BBI_). These findings indicate cardiovascular decoupling mediated through the sympathetic and parasympathetic system in order to allow concurrent blood pressure and HR increase.

In schizophrenia, we found a similar shift in sympathovagal balance and a reduction of cardiovascular coupling. HR and blood pressure were increased drastically. In the same line of thought, cardiovascular decoupling may be responsible for a missing mutual compensation of HR and blood pressure. The loss of sensitivity of baroreceptors might contribute to the pathologically impaired regulation. Similar to physical stress, mental load has been demonstrated to cause vagal inhibition as an adaptational mechanism in healthy controls (Bär et al., 2007a). Mental stress in acute schizophrenia might cause autonomic changes, like cardiovascular decoupling and, thus, tachycardia and hypertension. Indications of pathological changes of central regulation in schizophrenia include reduced activity of the medial prefrontal cortex. A lack of inhibitory control over amygdala-mediated responses to arousal might cause central suppression of vagal cardiovascular modulation (Bär et al., 2007a).

JSD and CCE both evaluate the occurrence of baroreflex related patterns in heart rate and systolic blood pressure symbol series. The JSD outcome is a word distribution matrix that allows one to extract the synchronous occurrence of specific symbol patterns (e.g., symmetric and diametric), whereas CCE estimates a causal influence of SBP on BBI by evaluating cross-compressibility. In contrast, BRS quantifies the mean magnitude of the baroreflex reaction no matter how often the reflex is initiated throughout the recording. In healthy subjects at rest, CCE and JSD were correlated to one another, and neither was correlated to any HRV index. The symbolic measures are less sensitive to the amplitude of heart rate fluctuations. In resting condition, variability of heart rate might not be driven primarily by the influence of blood pressure, but rather by respiratory activity. In patients as well as controls during exercise, CCE_BRS_ correlated positively to vagal indices (RMSSD, BRS_b_, BRS_t_) and negatively to markers dominated by the sympathetic system (HR, LF/HF). This relationship supports our assumption that CCE_BRS_ measures vagal baroreflex control that is inhibited by sympathetic cardiac activity. Relevant literature suggests, that in addition to the baroreflex, the impact of breathing on HR was decreased in patients (Peupelmann et al., 2009; Schulz et al., 2015) and during exercise (Goodwin et al., 1972; Iellamo et al., 1999). In these conditions, the contribution of the baroreflex to HR regulation seems to be different from rest. We failed to find these correlations in either JSD index. This might be one reason why both JSD measures were not changed significantly during exercise. In contrast, CCE reflected the suppression of baroreflex regulation initiated by the metaboreflex. Considering the absence of correlation to compression entropy of symbolized heart beat intervals (Hc_Y_), we conclude that CCE_BRS_ is not driven by univariate symbol series' variability.

Some limitations should be addressed. The amount of 20 surrogate datasets per subject appears to be hardly enough to conduct statistical testing, but was a trade-off between practicality and informative value. So-called “significant” CCE results mean that CCE of the original data set was higher than CCE calculated on at least 19 of 20 surrogates (5% error). Parameters for CCE_BRS_ assessment were defined *a priori* by considering baroreflex dynamics. The influence of calculation settings on CCE results was investigated in terms of surrogate analysis. These parameters are not transferrable to other physiological systems and should be defined according to the investigated dynamics individually.

CCE estimates predictability of one coarse-grained time series by another. The occurrence of target symbol patterns in the source series increases CCE, but only if compression by the target's own past is enhanced. Improving compressibility can be interpreted as an increase of predictability of the target's future by the source series, beyond the target's history, which is in compliance with the principle of causality formulated by Wiener and Granger (Wiener, 1956; Granger, 1969; Bressler and Seth, 2011). The direction of coupling is implicated by stating a minimum delay of redundant symbol patterns. Although a lag of zero theoretically does not represent a directed interaction, instantaneous influence involves physiologically meaningful baroreflex coupling (Ottesen and Olufsen, 2011; Faes et al., 2012). In future developments, cross-compressibility should be conditioned on additional time series. When analyzing the cardiovascular system, the opportunity to account for respiration is important. Respiratory sinus arrhythmia and baroreflex regulation dominate short-term heart rate variability and interact on the central and peripheral level. It was shown that respiratory sinus arrhythmia might lead to an overestimation of the causal influence of SBP on BBI (Porta et al., 2012).

Some interesting properties make the CCE subject worthy of further investigation. In contrast to most established interaction measures with adjustable but constant delay, CCE allows the delay of coupling to vary during the time of acquisition. This is because redundant symbol patterns at different positions in the source window (start *v*) and with different lengths (*n*) can contribute to CCE outcome. Although CCE is so flexible, it is easy to interpret as it is defined by the ratio of two lengths. *Post-hoc* analysis of *v* and *n* allows the identification of dominant delay and length of redundant patterns. In the current study, cardiovascular regulation across different time scales and different time lags could be quantified by a single number.

The method is easy to implement and robust to non-stationarities in the data under investigation. Analyzing symbolized time series is less sensitive to outliers and noise. By converting the input into a much smaller set of symbols, detailed information about the amplitude is removed. For example, every increase above *T*_*x*_ is converted into the same symbol, even if the amplitude is artificially high (outlier). Noise that is limited to the detailed information (amplitude smaller than *T*_*x*_), will be diminished after symbolization. There are numerous possibilities for how input time series can be translated into symbol sequences. Patterns of several consecutive samples may be used to encode the original signal (Berg et al., 2010; Schinkel et al., 2012; Müller et al., 2013). The symbolization procedure may also be modified to analyze the data on multiple scales (Costa et al., 2002; Baumert et al., 2012, 2013b). In general, the more symbols are used in the transformation, the better the symbol series resembles the time series. Here, the choice of three different symbols implies substantial coarse-graining of the input series. Besides fixed amplitudes (*T*_*BBI*_ = 5 ms and *T*_*SBP*_ = 1 mmHg), thresholds can be defined with regard to the time series' variance (e.g., multiples of standard deviation). This approach is more adaptive to the underlying variability. In this study, we attempted to separate minor fluctuations from significant changes of blood pressure and heart rate. It appeared obvious to apply thresholds approved in the context of baroreflex analysis. Although compression entropy of symbolized BBI was different in study 1 but almost identical in study 2, we found CCE_BRS_ differences in both studies. Thus, we believe there is no bias of CCE outcome due to the symbolization itself. But the symbolization scheme has an influence on compressibility estimates and should be designed with regards to the investigated issue.

In conclusion, we have demonstrated the suitability of CCE for estimating coupling of cardiovascular time series. CCE is sensitive to changes induced by exercise and revealed pathological impairments in patients with schizophrenia. Our data suggest that CCE is able to quantify interactions in short, noisy, and non-stationary data.

## Author contribution

AS: Design and conception of the study, analysis and interpretation of the data, preparing the manuscript; StS: Analysis of the data, preparing the manuscript, critical revision; AV: Critical revision; SuS: Acquisition of the data; MB: Critical revision; KB: Study conception, preparing the manuscript, critical revision.

## Funding

The study was financed by internal funds only.

### Conflict of interest statement

The authors declare that the research was conducted in the absence of any commercial or financial relationships that could be construed as a potential conflict of interest.
